# It is safe to use the ulnar length difference to correct the radial length difference in the 3D-planning process of a radius osteotomy in patients with a distal radius malunion

**DOI:** 10.1186/s13018-024-05012-3

**Published:** 2024-08-30

**Authors:** Camiel J. Smees, Koen D. Oude Nijhuis, Stein van der Heide, Judith olde Heuvel, Job N. Doornberg, Anne J.H. Vochteloo, Gabriëlle J.M. Tuijthof

**Affiliations:** 1 Centre for Orthopaedic Surgery OCON, Hengelo, The Netherlands; 2 Department of Biomechanical Engineering, University of Twente, Enschede, The Netherlands; 3 Department of Orthopaedic Surgery, University Medical Centre Groningen, Groningen, The Netherlands

**Keywords:** Forearm length difference, Malunion, Ulnar length, 3D models

## Abstract

**Background:**

A corrective radius osteotomy is often performed in patients with a symptomatic distal radius malunion. In 3D-planned osteotomies, the unaffected radius is mirrored over the malunited radius after adjusting for left-right length differences using both ulnae. This approach assumes that ulnar length differences in a malunion population are similar to those in a healthy population. This study was conducted to analyze the difference in ulnar length in a distal radius malunion population and to assess the potential influence of age, sex, or malunion side on this difference.

**Methods:**

We evaluated 65 adult patients with distal radius malunion using bilateral forearm CT scans. 3D models of both ulnae were constructed, and length differences were determined along a standardized length axis. The results were compared to two populations without a radius malunion.

**Results:**

The average absolute ulnar length difference was 2.57 mm (SD 1.81), which was comparable to the two healthy populations. This difference was not significantly affected by age, sex, or malunion side.

**Conclusion:**

This study demonstrated that using the ulnar length difference to correct for radial length difference in the current 3D planning process, before using the contralateral radius as a template for a corrective osteotomy in patients with radius malunion, is safe.

**Supplementary Information:**

The online version contains supplementary material available at 10.1186/s13018-024-05012-3.

## Introduction

Secondary displacement of distal radius fractures initially treated with closed reduction and cast immobilization occurs in 36–64% of all patients [1, 2]. If not recognized and treated in time, such displacement results in a malunion of the distal radius. Malunion can also occur after primary plate fixation, with rates reported as high as 35% [3]. Pain, limited range of motion, and reduced grip strength are common problems for patients with a distal radius malunion [4–6]. Patients with a symptomatic distal radius malunion can be treated with a corrective osteotomy.

To ensure more precise correction of the radius, three-dimensional (3D) planned patient-specific cutting and drilling guides can be used in surgery. In 3D planning, the unaffected contralateral radius is considered the best anatomic representation of the pre-fractured shape of the malunited radius, thus serving as the intended shape to achieve with the osteotomy. The unaffected side is mirrored and projected over the affected radius. However, previous studies have shown that in a healthy population, a substantial difference in radial length between both arms exists [7, 8].

Therefore, before comparing the radii, a correction for the radial length difference must be performed. A study by Vroemen et al. found a strong linear relationship between intra-person length differences in the radii and ulnae [8]. Thus, by comparing the ulnar lengths of both arms, the radial length difference can be corrected: the difference in ulnar length is used to adjust the length of the radii. Thereafter, the 3D comparison of the healthy and malunited radius can be made, and the corrective osteotomy planned.

In a population of healthy individuals, Vroemen et al. and Hong et al. found a substantial difference in left-right ulnar length: 2.08 mm (SD 2.33) and 2.54 mm (SD 1.88), respectively [7, 8]. The current assumption in 3D analysis is that this ulnar length difference in a healthy population is also representative of a population of malunion patients. Clinically, the ulnar length is not typically affected by a distal radius malunion. However, this has never been proven. If the ipsilateral ulna is indeed affected by the radius malunion, using the ulna to correct for length differences in the radius will result in insufficient correction, and an alternative method must be used.

The primary goal of this study is to demonstrate that the current clinical practice of using the ulnar length difference to correct the length difference of the radii in the 3D planning process of a corrective osteotomy of a radius malunion is safe. Therefore, an analysis of the left-right difference in ulnar length in a distal radius malunion population, both as an absolute and a relative number, is performed and compared to the data of healthy populations reported by Vroemen et al. and Hong et al. [7, 8]. Secondarily, the potential influence of age, sex, or the side of radius malunion (dominant vs. non-dominant hand) on the ulnar length difference was analyzed.

## Methods

For this diagnostic imaging study, approval was obtained from the board of directors of OCON, and based on national criteria, medical ethics consent was waived.

### Patients

In this analysis, we included all adult patients (aged > 18 years) who were treated with a 3D-guided corrective osteotomy for symptomatic distal radius malunion between February 2015 and September 2022 from our prospectively collected cohort of corrective osteotomies. All patients had a bilateral computed tomography (CT) scan of the forearm preoperatively. Patients with forearm pathology other than distal radius malunion, those with bilateral pathology, and those with any previous forearm surgery were excluded. Demographic characteristics including age, sex, malunion side and dominant handedness were collected for all included patients.

### CT scans and segmentation

The following CT scan settings were used when obtaining the CT scans: A slice thickness of 0.6 mm, a B60 kernel and a matrix of 512 × 512. The voltage used was 120 kV, and the tube current was automatically regulated. The CT scans were performed with the patient in prone position with both arms overhead and palms facing each other.

Both forearms were segmented to create a 3D surface model using the 3D medical imaging processing software Mimics (Materialise, Leuven, Belgium). The ulnae were segmented with a threshold value of 300 Hounsfield units. A combination of region-growing, mask-splitting and hole-filling functions was used to obtain the 3D surface models of the ulnae. The segmentation method was previously validated and considered to be accurate [9]. All segmentations were performed by a 3D specialist (CS).

### Measurements

**Fig. 1:**
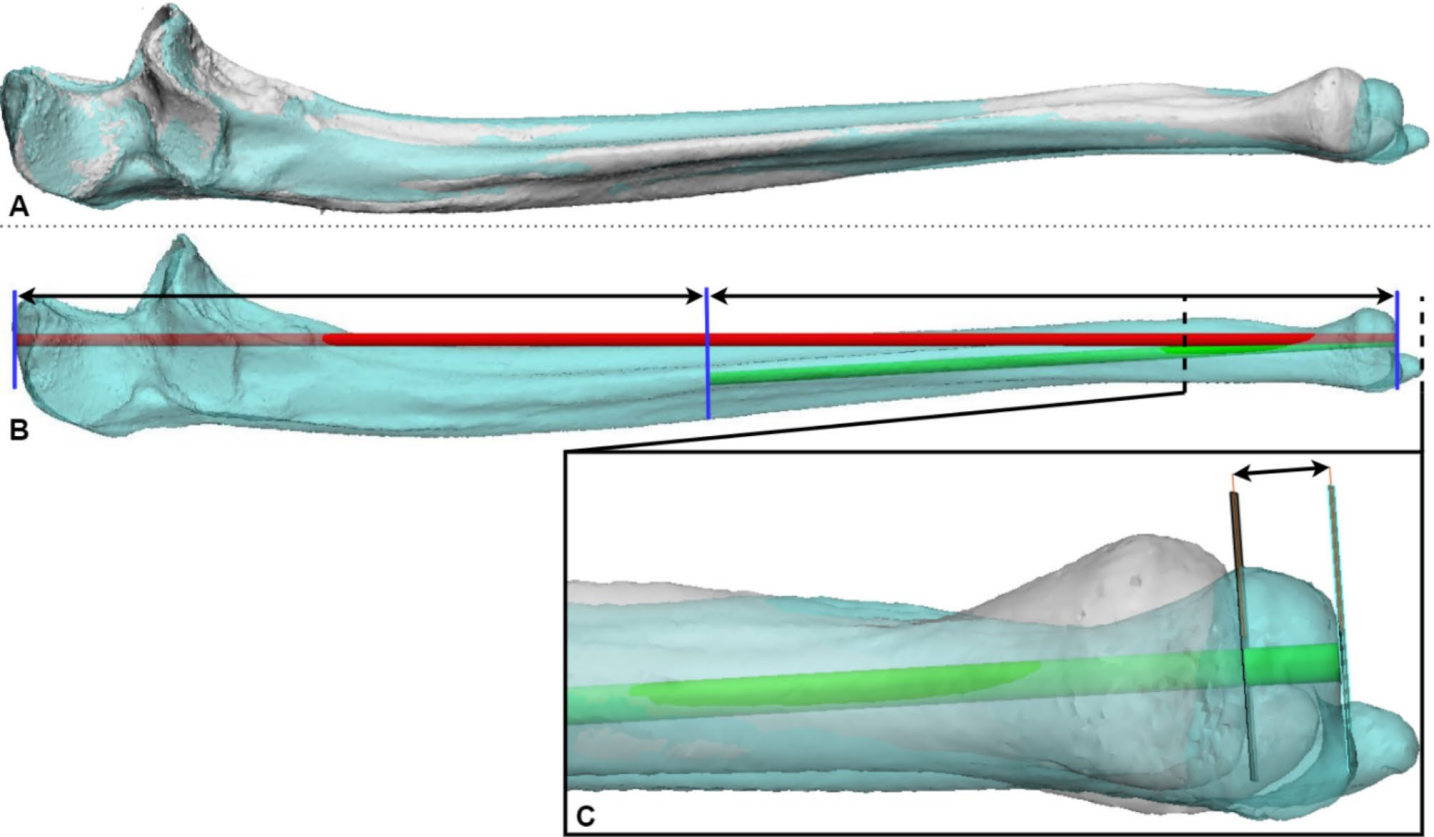
Measuring the ulnae length difference. **A**) The left ulna (cyan) is mirrored and aligned with the contralateral ulna (white). The longest ulna is then determined. **B**) On the longest ulna, the halfway point is defined as the point between the tip of the olecranon and the ulnar dome (red). The length axis (green) is defined between the halfway point of the ulna and the tip of the ulnar dome. **C**) The ulnar length difference is measured on the length axis between the two ulnae

The first step in carrying out the measurements is creating a standardised measurement axis. The definitions from the International Society of Biomechanics (ISB) were followed as closely as possible[12]. First, the left ulna was mirrored and aligned with the right ulna from proximal to distal, through a combination of semi-automatic matching and manual adjustment (Fig. 1a). Matching was deemed successful if the olecranons overlapped and the ulnar domes were aligned. The longest of the two ulnae was used to determine the standardised measurement axis. In the longer ulna, the ulna is cut halfway between the olecranon and the tip of the ulnar dome (Fig. 1b). To determine the centre of the cut surface, an arc was fit to this surface. The measurement axis was formed between the middle of this arc and the tip of the ulnar dome (green line in Fig. 1b). The length difference was measured along this axis as a difference between the heights of the ulnar domes (Fig. 1c). These measurements were taken by a junior researcher (SvdH) and a 3D specialist (CS). In case of a measurement discrepancy of more than 1 mm, another 3D specialist was consulted who decided which of the two measurements was correct. A detailed explanation of the measurement protocol can be found in Appendix 1.

### Statistical analyses

A power analysis was performed to estimate the required patient population size to describe the difference in ulnar length in this cohort. [10] Two previous studies were used to determine a weighted average standard deviation of 1.95 mm with a normal distribution. [7, 8] With a desired confidence interval of 0.5 mm and a 5% two-tailed confidence interval, the calculated minimum sample size is 59.

To assess the reliability of the measurements, the mean inter-rater differences with standard deviation and the intraclass correlation coefficient (ICC) were calculated. The guidelines for interpretation of the ICC given by Koo and Li were used as standards. [11]

Normality of all groups was verified using the Kolmogorov–Smirnov test. All variables were normally distributed. The main outcome of this study was the mean and standard deviation of the absolute length differences of the ulnae. The ulnar length difference relative to the full ulnar length, expressed as a percentage, was also calculated. T-tests were performed to assess whether significant differences in bilateral length differences (absolute and relative) were present in groups based on age, sex and malunion in the dominant vs. non-dominant side. Age groups were defined as 18–49 and 50 + years old. Statistical significance was set at *p* < 0.05.

## Results

### Demographics

Sixty-five patients were included. The mean age of the population was 47 years (SD 17), 75% was female, and 52% had a malunion of the non-dominant wrist (Table 1).

**Table 1:** The demographics of the included population. The mean absolute and relative ulnar length differences are given for the entire population, as well for the group divided by age, sex and fracture side and the corresponding *p*-value

Variables	*N* (%)	Absolute ulnar length difference		Relative ulnar length difference	
		Mean (SD) (mm)	*p*-value	Mean (SD) (%)	*p*-value
N	65 (100%)	2.57 (1.81)		0.96 (0.66)	
Age	47 ± 17		0.38		0.40
18–49 years	33 (51%)	2.71 (2.07)		1.03 (0.75)	
50 + years	32 (49%)	2.31 (1.51)		0.89 (0.55)	
Sex			0.27		0.59
Male	16 (25%)	2.95 (2.17)		1.04 (0.75)	
Female	49 (75%)	2.37 (1.68)		0.94 (0.63)	
Malunion side			0.62		0.63
Dominant side	31 (48%)	2.63 (1.74)		1.00 (0.62)	
Non-dominant side	34 (52%)	2.41 (1.89)		0.92 (0.70)	

### Intraclass correlation coefficient

Two outliers with more than a 1 mm difference were checked by a third observer. The ICC between both observers was always above 0.99, both before and after the two outliers were checked, thus indicating excellent reliability. [11]

### Absolute ulnar length differences

**Fig. 2:**
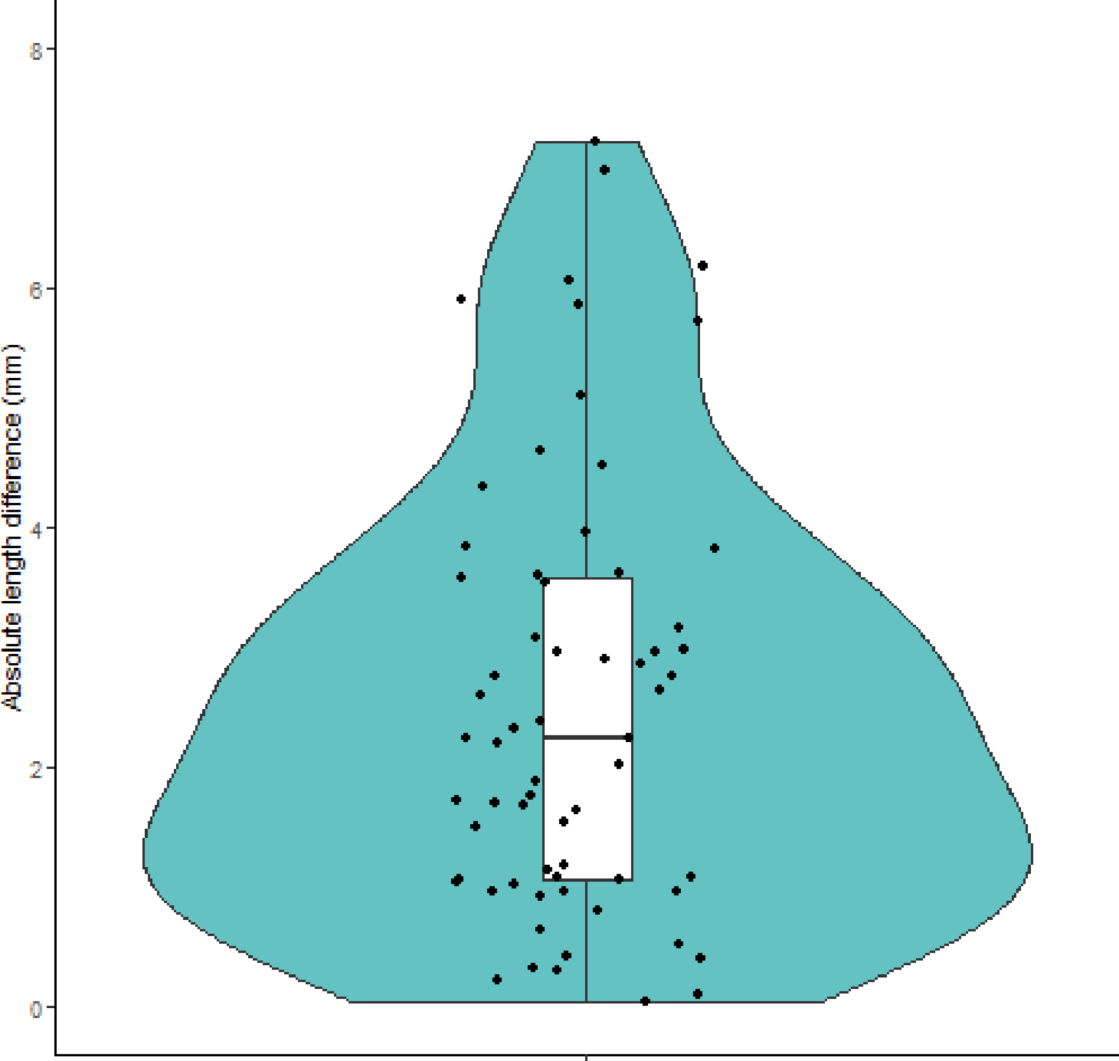
The spread in absolute bilateral length differences. The violin plot depicts the absolute length differences in the entire population as mean values of both observers. Every dot represents the measurement in one patient. The mean length difference is 2.57 mm (SD 1.81)

The measured mean absolute length difference in this population was 2.57 mm (SD 1.81). The right arm was, on average, 1.02 mm (SD 2.98) longer than the left arm. Absolute length differences were not significant among groups divided by age, sex, and whether the malunion was present in the dominant or non-dominant side (*p* = 0.38, 0.27, and 0.62, respectively; see Table 1). Figure 2 presents a violin plot of the absolute length differences, with the minimum difference found being 0.05 mm and the maximum difference being 7.23 mm.

### Relative bilateral ulnar length differences

The mean of the relative length differences in the total study population was 0.96% (SD 0.66). There were no significant differences in relative length differences among groups divided by age, sex and malunion present in the dominant vs. the non-dominant hand: *p* = 0.40, 0.59 and 0.63, respectively (Table 1).

## Discussion

In this paper, we demonstrate that the common practice in 3D planning of a corrective osteotomy - using the ulnar length difference to correct the radial length difference in cases of radius malunion - is safe. This conclusion is based on measurements of the length difference between the left and right ulna in a population with distal radius malunion and comparisons of this difference to previous studies on patients without forearm pathology. We found a length difference of 2.57 mm (SD 1.81), comparable to those in two populations without forearm pathology: 2.54 mm (SD 1.88) and 2.08 mm (SD 2.33) [7, 8]. This finding suggests that a distal radius malunion has little to no effect on the ipsilateral ulna, and that the small recorded differences may result from variations in measurement strategies and population differences.

In our study, we aligned the ulnae from proximal to distal and determined the length difference along the length axis, constructed based on ISB guidelines. In contrast, Hong et al. calculated the lengths of both ulnae separately within their own coordinate systems before determining the differences [8, 12]. Vroemen et al. did not specify the coordinate system used for their ulnar measurements [7].

In addition to lengthwise asymmetry measured in this study, both Vroemen et al. and Hong et al. also included asymmetry measurements in different axes [7, 8]. However, they found that only lengthwise differences were significant. Because length is one of the most critical factors to correct for, we opted to consider only the lengthwise differences. Combined with the fact that our method aligns with ISB standards and this method demonstrated excellent intra-observer reproducibility, we propose that future studies should consider methods similar to ours. Appendix 1 describes a detailed step-by-step guide to perform the length measurements.

This study assessed a Dutch population. Vroemen et al. also used a Dutch cohort, while Hong et al. studied a Korean population [7, 8]. Given the similarity of results between these studies, we expect our findings to be generalizable to other populations.

Bilateral length differences (absolute and relative) were not significantly different among groups based on age, sex, or side of radius malunion (dominant vs. non-dominant). The population size for the power analysis was based on the mean bilateral length difference, not on subanalyses. Post-hoc power calculations for the subanalyses revealed insufficient population sizes for these analyses. It is therefore unclear whether age, sex, or side of radius malunion significantly affects bilateral length differences in a larger population.

In addition to the studies by Hong et al. and Vroemen et al., we presented relative length measurements, considering the expectation that taller individuals might have longer forearms and that significant height differences may exist among different populations [7, 8]. However, this percentage was very small, rendering this assumption irrelevant.

### Limitations

Our study verified the importance of correcting for left-right length differences. However, the maximum bilateral length difference for which a simple length correction is sufficient and the threshold at which alternative methods, such as statistical shape modeling, should be used remain unknown. Additionally, this study analyzed only ulnar length, as malunion was present in one of the radii. It is expected that ulnar length is a useful indicator of radial length, as previously demonstrated by Vroemen et al. [8] However, the linear relationship identified by Vroemen et al. between inter-person length differences in the radii and ulnae is currently based on 20 healthy volunteers and is assumed to hold for all patients. In the population of Vroemen et al., the largest reported length difference in the ulnae was around 6.7 mm, while in our population the largest length difference was 7.30 mm. It is unclear whether the linear relationship reported by Vroemen et al. is maintained when length differences exceed the currently observed range. To gain a better understanding of the relationship between radial and ulnar lengths and potential variations, further investigation in a larger healthy population is necessary.

### Clinical implication

This study found that the ulnar length differences in a distal radius malunion population are comparable to those in a healthy population. The current workflow of correcting for forearm length differences using the ulna is therefore also verified in a malunion population.

Because correcting for forearm length differences has been deemed an important step in preparation for a radial osteotomy, these differences are often measured. In this study, the measurements were taken manually, specifically to ensure that the steps are easily reproducible and automatable. Appendix 1 contains a detailed protocol of all steps, which can be used to automate the measurements described in this paper.

## Conclusion

The substantial difference between the left and right ulna in patients with distal radius malunion that we found was similar to that in people without forearm pathology [7, 8] Previous research has shown a strong correlation between intra-patient ulnar and radial left-right length differences [8]. Therefore, this study demonstrated that it is safe to use the left-right length difference of the ulna to correct for the radial length difference in 3D-planned corrective osteotomy of a radius malunion, which uses the contralateral radius as a template.

### Electronic supplementary material

Below is the link to the electronic supplementary material.

Additional file 1: Appendix 1, Measurement protocol. Automated length measurements using Materialise 3-Matic

## Data Availability

The datasets used and/or analysed during the current study are available from the corresponding author on reasonable request.

## Abbreviations

3D: Three–dimensional
CT: Computed Tomography
ISB: International Society of Biomechanics
ICC: Intraclass correlation coefficient
