# Supplementary material for: It is safe to use the ulnar length difference to correct the radial length difference in the 3D-planning process of a radius osteotomy in patients with a distal radius malunion
Source: J Orthop Surg Res. 2024 Aug 30;19:525. doi: 10.1186/s13018-024-05012-3 (PMC11363621; doi:10.1186/s13018-024-05012-3)
Supplement: Supplementary file 1 — Additional file 1: Appendix 1, Measurement protocol. Automated length measurements using Materialise 3-Matic [file 13018_2024_5012_MOESM1_ESM.pdf]

Appendix I, Measurement protocol

Automated length measurements using Materialise 3-Matic

The following protocol describes a series of steps to measure ulnar length differences. The proposed steps can be automated within 3-Matic, with the exception of step 2. The ISB guidelines were followed regarding the definition of the coordinate system of the ulna (Wu et al., 2005).

In general, three parts are performed: aligning the models, constructing the length axis and performing the measurement. The steps of aligning the models and constructing the length axis are intertwined and therefore combined in the first section.

Aligning the models and obtaining the length axis

1. Load the 3D models of a pair of ulnae.
2. Manually mark the ulnar heads (without the styloid) of both ulnae.
3. Fit one plane to the left and right ulna models combined.
4. Mirror the left ulna using the XZ-plane of the created plane.

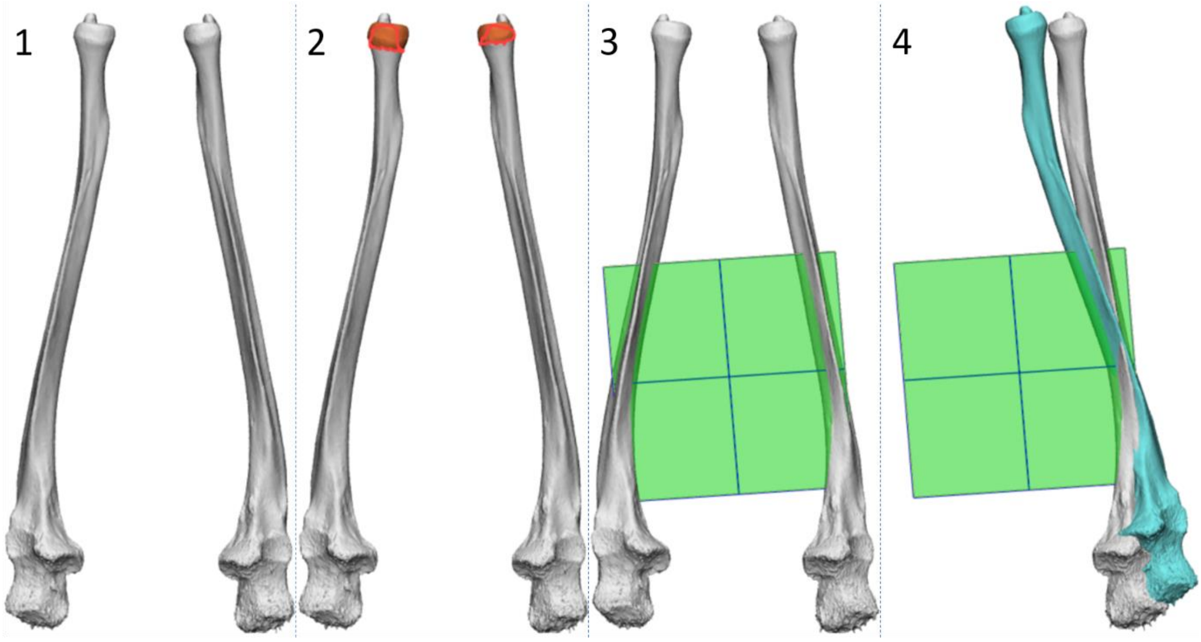

For both ulnae:

5. Convert the marked surface to a mesh.
6. Fit a sphere separately to each ulnar head mesh.

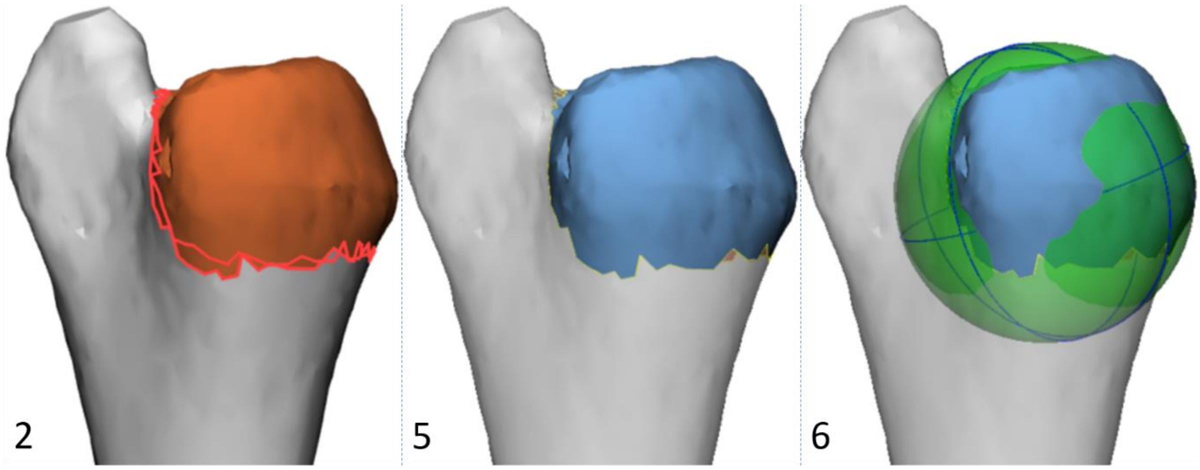

7. From the sphere, the distance to the corresponding ulna is determined.
8. The maximum 1% of distances found is assumed to be the most proximal region of the olecranon.
9. Determine the average point of this region.
10. An axis between the centre of the sphere and the proximal point is determined.
11. This axis is proximally and distally extended until it intersects the ulnar surface to determine the proximal and distal ends of the axis.

Continue generally:

12. The proximal points are used to overlap the left ulna with the right ulna proximally.
13. The difference in direction of the axis is then used to determine the direction of the distal part.
14. Global registration is then used to further improve the alignment.
15. Visually verify the alignment.

16. Using these lines from both ulnae, the longest of the two ulnae is determined.
17. A plane is created in the middle of the line of the longest ulna, perpendicular to this line.
18. This plane is used to cut the longest ulna halfway.

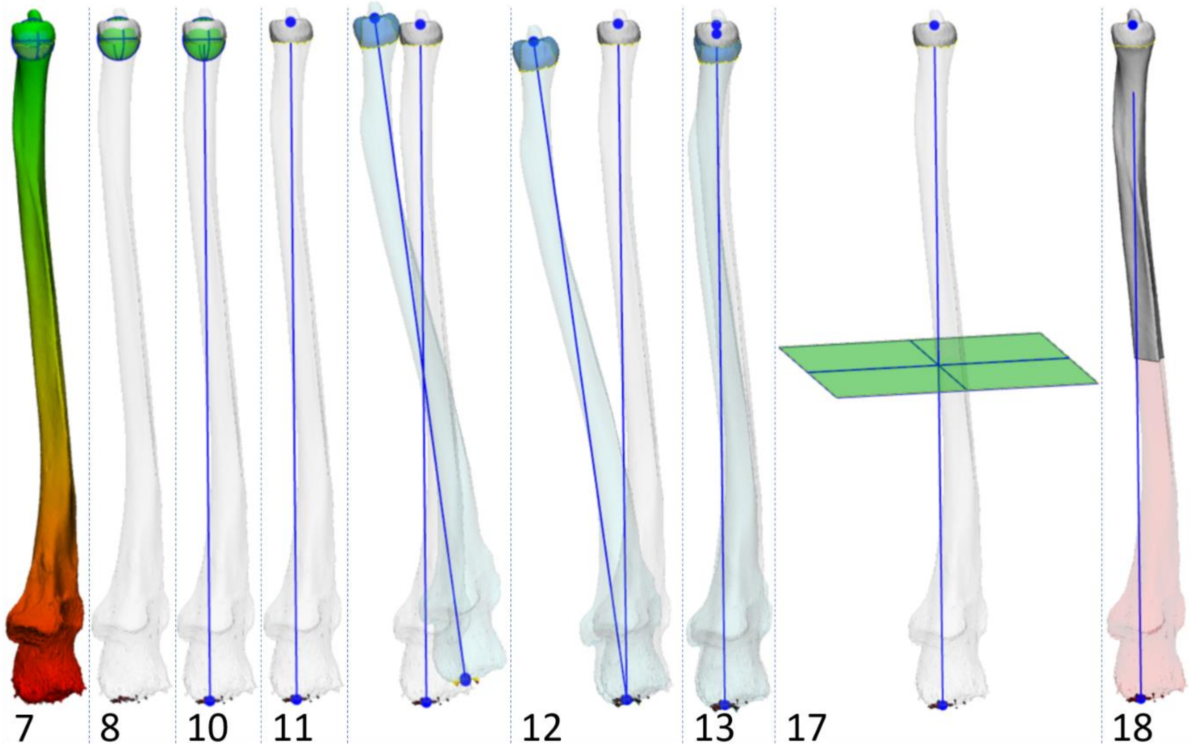

19. On the cut surface, an arc is fit, that best fits the cut surface.
20. A new line is created from the centre of the arc to the previously created sphere, this line represents the direction of the length axis.

Measurements

Now that the direction of measuring is determined, the measurements can be performed.

21. For each ulna separately, perform an extrema analysis. Use the predetermined length axis as measurement direction, and determine the global maximum of the ulnar head surfaces.
22. Determine the distance in the length direction between both maxima.

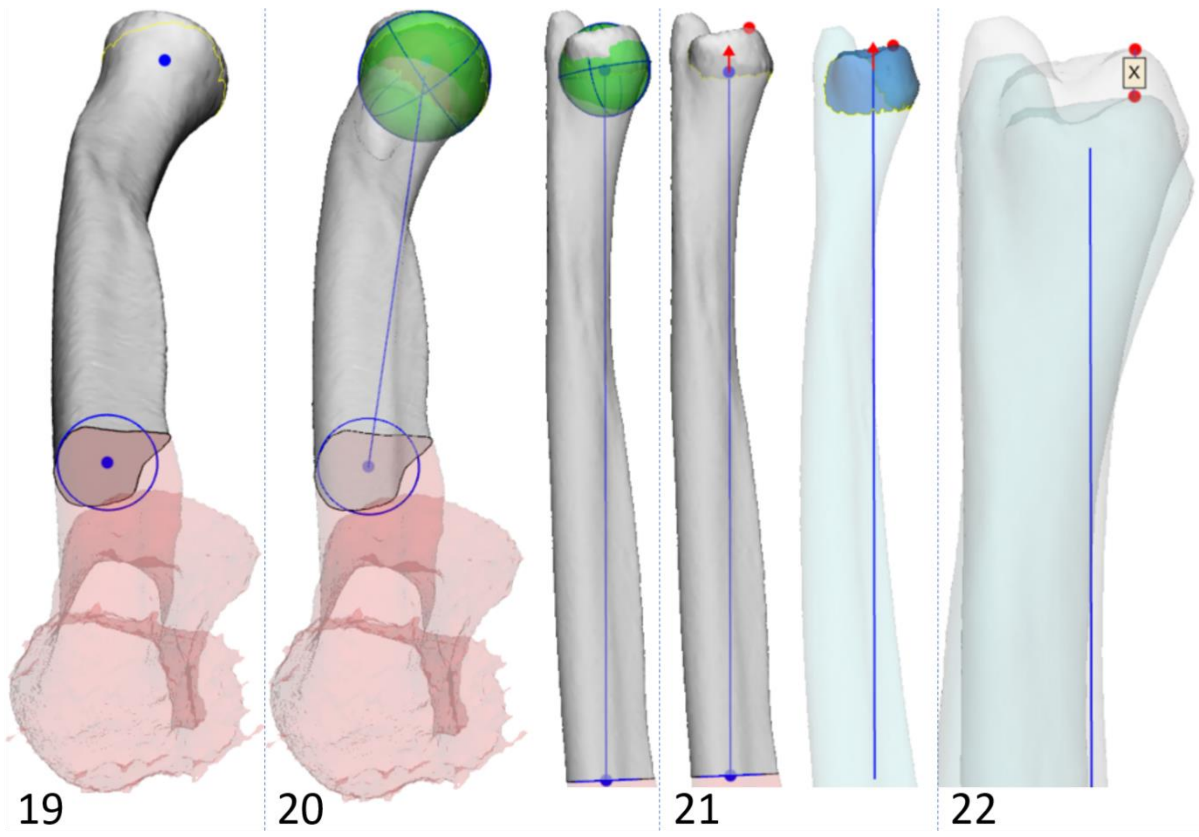
19
20
21
22
